# AZO-Based ZnO Nanosheet MEMS Sensor with Different Al Concentrations for Enhanced H_2_S Gas Sensing

**DOI:** 10.3390/nano11123377

**Published:** 2021-12-13

**Authors:** Yempati Nagarjuna, Jun-Cong Lin, Sheng-Chang Wang, Wen-Tse Hsiao, Yu-Jen Hsiao

**Affiliations:** 1 Department of Mechanical Engineering, Southern Taiwan University of Science and Technology, Tainan 710, Taiwan; arjunrocky786@gmail.com (Y.N.); ma810116@stust.edu.tw (J.-C.L.); scwang@stust.edu.tw (S.-C.W.); 2 Taiwan Instrument Research Institute, National Applied Research Laboratories, 20 R&D Road VI, Hsinchu Science Park, Hsinchu City 300, Taiwan

**Keywords:** H_2_S gas sensing, ZnO nanostructure, MEMS device, AZO, gas selectivity

## Abstract

The properties of H_2_S gas sensing were investigated using a ZnO nanostructure prepared with AZO (zinc oxide with aluminium) and Al surfaces which were developed on a MEMS (Micro Electromechanical System) device. Hydrothermal synthesis was implemented for the deposition of the ZnO nanostructure. To find the optimal conditions for H_2_S gas sensing, different ZnO growth times and different temperatures were considered and tested, and the results were analysed. At 250 °C and 90 min growth time, a ZnO sensor prepared with AZO and 40 nm Al recorded an 8.5% H_2_S gas-sensing response at a 200 ppb gas concentration and a 14% sensing response at a gas concentration of 1000 ppb. The dominant sensing response provided the optimal conditions for the ZnO sensor, which were 250 °C temperature and 90 min growth time. Gas sensor selectivity was tested with five different gases (CO, SO_2_, NO_2_, NH_3_ and H_2_S) and the sensor showed great selectivity towards H_2_S gas.

## 1. Introduction

H_2_S gas is one of the most harmful gases, and it can have diverse effects on the environment and human health. H_2_S is a colourless toxic gas with a rotten or stinky smell, and it has been considered as one of the critical endogenous gasotransmitters [1,2]. H_2_S gas is a by-product of different industries such as petroleum refining, sewage systems, aquaculture and natural gas production [3,4]. Due to industrial usage, every year, 3 million tons of H_2_S gas are released into the environment [5]. Exposure to H_2_S gas can lead to many health problems such as eye irritation, and throat and respiratory problems [6]. At low-concentration exposure levels, such as 2 ppm, health effects including nausea and loss of sleep may occur, and at high concentrations such as 50 to 100 ppm, adverse health effects including respiratory tract irritation, digestive problems and loss of appetite may occur [7]. Due to all these reasons, there is a need for the detection of H_2_S gas at low-level gas concentrations.

Metal oxide semiconductors have been widely used for sensing applications including gas sensors [8], glucose sensors [9], sweat sensors [10], etc. because of the properties that semiconductors possess, such as large specific surface areas, mechanical strength and excellent gas permeability [11]. Some of the most used metal oxide semiconductors include ZnO [12], SnO_2_ [13], WO_3_ [14], NiO [15], etc. and, among them, many efforts have been undertaken on ZnO to promote its gas-sensing properties.

ZnO is a promising metal oxide n-type semiconductor for gas sensing, with a wide range of properties including a large specific surface area, high electro mobility and good photoelectric response, and it also has a wide band gap of 3.37 eV and 60 meV excitation binding energy [16]. ZnO material also has advantages such as low production cost, good physical and chemical properties, and no produced pollution [17]. To enhance the sensing properties of a particular sensor, metal oxide semiconductors such as ZnO are sometimes doped with noble metals including Au and Pt [18] and enhancement can also be achieved via the mixing of two metal oxides, for example, ZnO/CuO [19]. The sensing properties of ZnO depend upon the variety of structural morphologies and shapes from the structures, including zero-dimensional nanoparticles (0D), one-dimensional rods (1D), two-dimensional sheets (2D) and three-dimensional hierarchical structures (3D) [20]. Among the different nanostructures, thin films of ZnO are said to exhibit high degrees of gas sensitivity, mainly because of their polycrystalline nature and large exposed surface area [21]. Thus, when a metal oxide gas sensor comes into contact with a reducing gas or an oxidizing gas, there will be a noteworthy change in the resistance value of the sensor [22]. This change in resistance will help in finding the gas-sensing response of the particular gas sensor.

Synthesis of ZnO nanoparticles can be achieved using physical, chemical and biological methods. Each of these methods has different techniques that can be used to prepare different types of nanoparticles depending upon the process parameters. Chemical synthesis methods include the sol–gel process, as well as hydrothermal, solvothermal, precipitation and microemulsion approaches. Some of these processes can synthesise nanoparticles at low temperature and some may require high-temperature and high-pressure conditions, which may lead to high energy consumption. Physical methods include physical vapor deposition, the arc plasma method and ultrasonic irradiation. These physical methods include robust equipment with high energy inputs; thus, these techniques involve high production costs. Biological methods include green synthesis and biotechnology. Biological methods do not require toxic chemicals for the synthesis process and it’s completely eco-friendly and requires low-cost production since this process does not require high energy inputs [23,24].

In the current study, a ZnO nanostructure is prepared using hydrothermal synthesis with different growth times. Among the different nanostructure deposition methods, many researchers opt for hydrothermal synthesis because of its simple operation, low cost, mild reaction conditions and high product purity [25]. The hydrothermal approach used in the aforementioned research is implemented at 90 °C in a micro-oven and does not involve any end products that are harmful for nature; therefore, the ZnO preparation process is an environmentally friendly approach. In the current study, the ZnO structure was prepared on a MEMS device to assess its gas-sensing properties and the MEMS device was also studied in terms of its thermal properties to ensure the high quality of the sensing property results. ZnO was developed over AZO and Al substrates, which were deposited using the ion-sputtering process. H_2_S’s gas-sensing properties were investigated with different concentrations under different temperature conditions. A minimum concentration of 200 ppb and a maximum concentration of 1000 ppb were tested in the current study.

## 2. Materials and Methods

### 2.1. MEMS Device Fabrication

**Figure 1:**
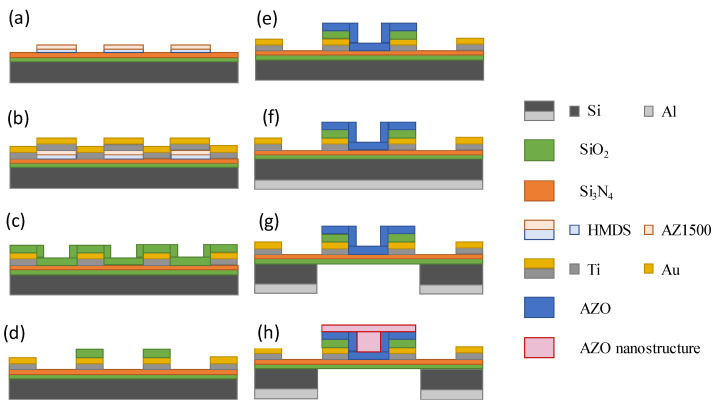
(**a**–**h**): Fabrication process of the MEMS microheater device.

The fabrication of the MEMS device, which is shown in Figure 1, started with the cleaning of the silicon wafer (of 400 µm size) with acetone and isopropanol. Then, layers of SiO_2_ and Si_3_N_2_ were deposited on the silicon wafer substrate. A photoresist layer was added to the surface (HMDS AZ1500) to define the electrode pattern according to the first mask exposure, as shown in step (a), and an electron gun (E-Gun) was used to deposit gold (Au) and titanium (Ti) electrodes, as shown in step (b). HMDS (Hexamethyldisilazane) was used in the photoresist process as a base layer for the MEMS device used in the spin coating process at a speed of 500 rpm. After HMDS, AZ 1500 series photoresist was added to the MEMS device with a thickness ranging from 0.4 to 0.5 µm at 2000 rpm. Finally, the lift-off method was used to finish the fabrication of the MEMS microheater and the sensing electrodes.

After completing the electrodes, next step was to deposit the insulating layer on the microheater. The photoresist coating was added onto the surface to complete the development of the second mask, and then silicon dioxide was deposited (SiO_2_) by plasma-assisted chemical vapor deposition (PECVD), as shown in step (c), and finally, the microheater was soaked in acetone to remove the photoresist on the surface in order to complete the insulating layer, as shown in steps (d) and (e).

For the sensing film, the photoresist needed to be coated on the surface before completing the exposure and development of the third mask and depositing aluminium zinc oxide (AZO) of 100 nm thickness on the surface using RF sputter, as shown in step (h). Finally, hydrothermal synthesis was used to develop the ZnO nanostructure with different growth conditions. A layer of aluminium was coated on the back of the silicon wafer for the back-etching process. A fourth mask was developed using the photoresist, and when the pattern was developed on the surface, acetone and aluminium etching solution were used to remove the photoresist, as shown in steps (f) and (g).

**Figure 2:**
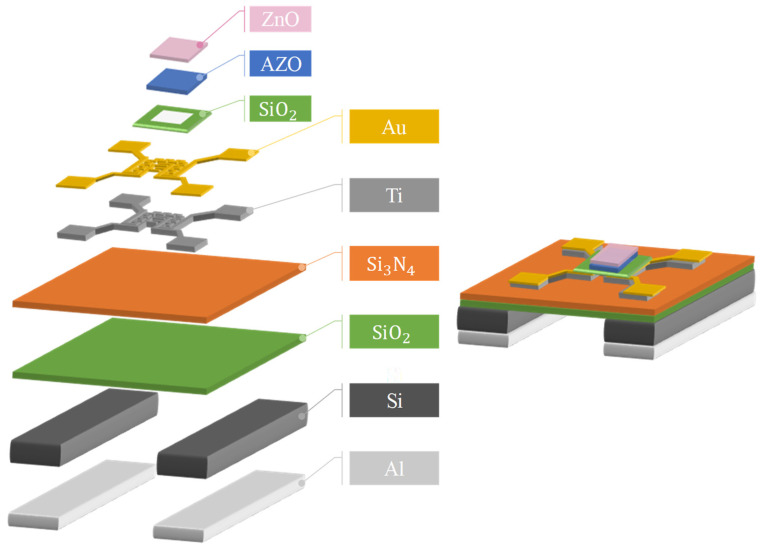
Three-dimensional figure of the fabricated MEMS microheater.

After the back-etching process was completed, the four electrodes of the microheater were coated with the photoresist to protect the electrodes before the hydrothermal process or wire bonding were begun. When all the processes were finished, the required amount of Al was deposited on the microheater for the development of ZnO nanostructure using hydrothermal synthesis. The finished 3D design product of the MEMS microheater device is shown in Figure 2.

### 2.2. ZnO Nanostructure Development

Thin ZnO films were developed over different thicknesses of Al such as 10 nm, 20 nm, 30 nm and 40 nm. To prepare the solution of ZnO, 0.5 g of Zinc Nitrate Hexahydrate (Zn(NO_3_)_2_·6H_2_O) and 0.5 g of Hexamethylenetetramine (HMTA or C_6_H_12_N_4_) were taken in a laboratory bottle and 100 mL of distilled water was added. Zinc Nitrate Hexahydrate (Zn(NO_3_)_2_·6H_2_O) of 98% purity was ordered from SIGMA-ALDRICH and Hexamethylenetetramine (HMTA or C_6_H_12_N_4_) of 99% purity was ordered from Alfa Aesar company (Haverhill, MA, USA). The compounds were stirred using a mixing hot plate for about 3 min and the Al-coated microheaters were dispensed in a laboratory bottle. Hydrothermal synthesis took place after the preparation of the ZnO solution. The bottle was placed in the micro-oven for heating at 90 °C for time periods of 45 min, 90 min, 180 min and 270 min. After the time periods had completed, the ZnO nanostructures were be deposited on the Al surface of the microheater. Hydrothermal synthesis was carried out in the CHANNEL DV302 model heating oven, which operated with an input supply of 110 Volts and with a maximum heating temperature of 200 °C. After the microheaters were removed from the oven, they were annealed at 500 °C to increase their crystalline nature. The annealing process was conducted for 30 min. Annealing was carried out in a SUNRISE M-1 high-temperature furnace system. With a power supply of 1500 watts and 110 Volts, this furnace can reach maximum of 0 to 1200 °C in 1 h. The annealing process was carried out in the presence of air. Then, the thin ZnO films/MEMS microheaters were used for the study of the structural and sensing properties.

Structural properties such as XRD graphs were assessed using a D8 Advance ECO X-ray diffractometer machine (Bruker co., Billerica, MA, USA). This machine has an angular range of 360° and with a power supply of Single phase 200 to 350 Volts. SEM images and cross-sectional images were obtained using a HITACHI SU8000 Scanning electron microscope (Hitachi co., Chiyoda City, Tokyo, Japan). This equipment has 30 to 2000× low magnification and 400 to 800,000× high magnification with an acceleration voltage of 0.1 to 2 KV. EDS spectrum analysis was also conducted with the same HITACHI SU8000 scanning electron microscope. The thermal properties of the MEMS device were examined using a high-performance CHCT p 384 series thermal image camera, which was connected to a MOSTECH LPS-305 power supply system. Thermal analysis was conducted using IRM-P384 software (CHCT co., LTD, Taipei, Taiwan) in which the voltage readings from the power supply determined the temperature of the sensor.

Gas-sensing measurements were conducted with the sensor being placed in a chamber and with the heating electrodes being connected to a MOSTECH LPS-305 power supply system. This power supply system has a maximum power output of 165 Watts with a maximum voltage of 3 Volts and a current of 2.5 Amperes. The sensing electrodes were connected to the KEITHLEY 2400 DC power supply source meter to obtain the I–V characteristics of the sensor. The I–V characteristics provided the gas-sensing response in relation to conductivity and resistance.

## 3. Results and Discussion

**Figure 3:**
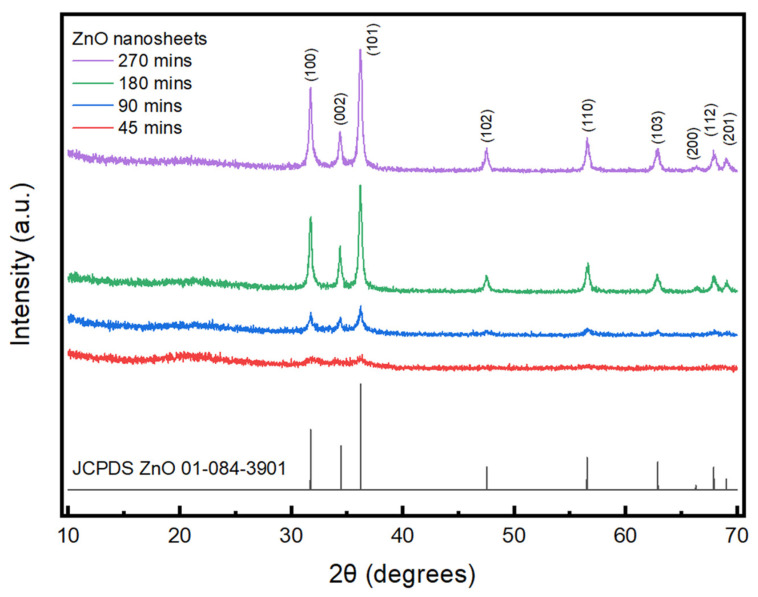
XRD image of ZnO nanosheets at different growth times.

The XRD graphs of ZnO nanosheets at different growth times, such as 45 min, 90 min, 180 min and 270 min, are shown in Figure 3. The ZnO samples mentioned in the graph were annealed at 500 °C. As the growth time increased, there was a distinguishable growth in the ZnO peaks. The peaks of ZnO included (100), (002), (101), (102), (110), (103), (200), (112) and (201) and all the peaks were in accordance with JCPDS ZnO 01-084-3901. There was a good agreement between the ZnO peaks obtained in the study and with the standard JCPDS structure. In the XRD data, there were no diffraction peaks, which proves that there was no presence of impurities. This indicates that the synthesis method of ZnO was successfully implemented with maximal purity levels.

The crystalline size can be calculated using the formula D = K λ/d cosθ. In this formula, K is a dimensionless shape constant with a value of 0.94. λ is the X-ray wavelength with a value of 1.54. d is the full width at half maximum (FWHM) in radians. θ is the Bragg angle. An example of the average crystal size calculation is provided for a 270 min deposition sample and the results are as follows. From the XRD graph, the d value of the peak is 0.331 radians. The Bragg angle is 36.21 radians. From the data gathered, the crystalline size is calculated to be 26.46 nm.

**Figure 4:**
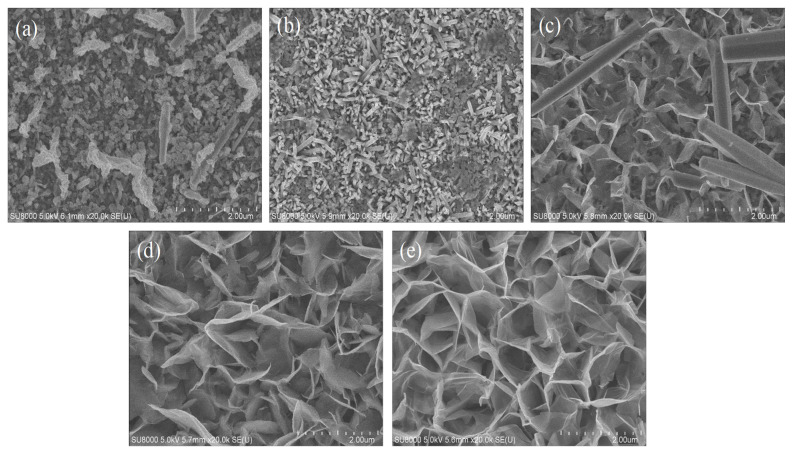
SEM images of (**a**) pure AZO, (**b**) AZO with 10 nm of Al, (**c**) AZO with 20 nm of Al, (**d**) AZO with 40 nm of Al, (**e**) AZO with 80 nm of Al.

The SEM images of different compositions of AZO with Al is shown in Figure 4a–e. The synthesis of ZnO using hydrothermal process varied with each concentration of Al content. In Figure 4a, ZnO is shown to be synthesized on the surface of pure AZO with no addition of Al content. Similarly, from Figure 4b–d, it can be seen that ZnO was synthesized on the surface of AZO with the addition of Al of different thicknesses, i.e., 10 nm, 20 nm, 40 nm and 80 nm, respectively. From the images, it is understood that the hydrothermal synthesis of ZnO required a minimum of 20 nm Al content. Figure 4a–c show that a kind of agglomeration was formed, and this phenomenon indicated that the synthesis process of ZnO formation was incomplete. On the other hand, Figure 4d,e shows the formation of a good structure with pores and a web-like pattern. The pores’ presence was effective during the assessment of gas sensing in the process of adsorption. Since the AZO element contains aluminium, instead of an 80 nm aluminium thickness, the current study favoured the inclusion of AZO with a 40 nm aluminium thickness in order avoid large amounts of aluminium being present in the nanostructure. All the samples mentioned had a growth time of 45 min.

**Figure 5:**
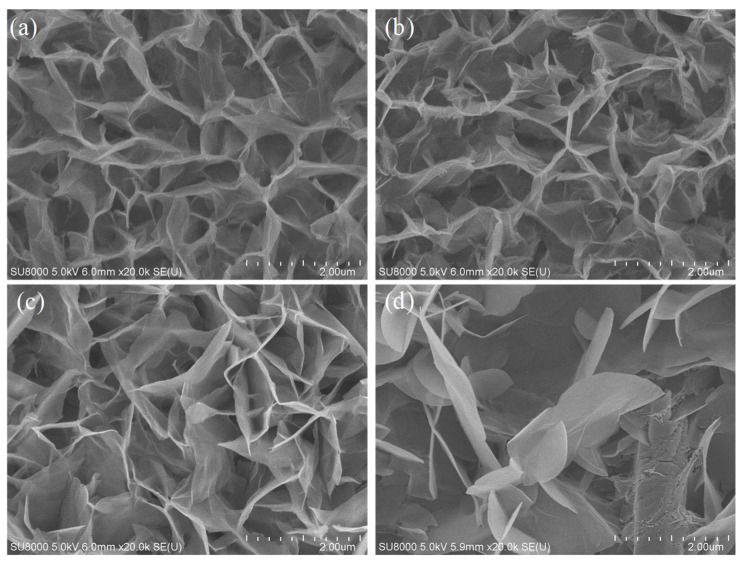
Comparison of ZnO nanostructure prepared on 40 nm Al AZO with different growth times: (**a**) 45 min, (**b**) 90 min, (**c**) 180 min, (**d**) 270 min.

SEM analysis was carried out to find out the surface morphology of the ZnO nanostructures with different growth times. The growth of the ZnO nanostructure on AZO with 40 nm Al is shown in Figure 5 with different growth times. The growth of the ZnO nanostructure was undertaken at 45 min, 90 min, 180 min and 270 min, respectively. For the composition of AZO with 40 nm Al, the deposition of ZnO was smooth at all the different growth times and ZnO was deposited uniformly all over the surface. Structural morphology analysis of all samples was performed at the same magnification levels to differentiate the structural properties from the synthesis at different growth times. SEM images were synthesised at 45 min and 90 min growth time, and showed high ZnO structural content consisting of large amounts pores and a web structure. SEM images recorded at 180 min and 270 min growth time showed the formation of small agglomerations with uneven ZnO formation. All of the samples mentioned in the figure were annealed at 500 °C.

**Figure 6:**
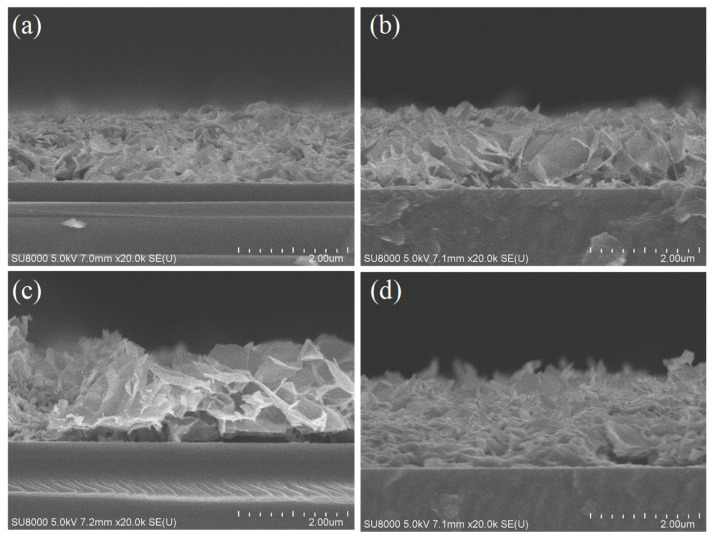
Comparison of ZnO nanostructure cross-sectional SEM images prepared on 40 nm Al AZO under different growth times (**a**) 45 min (**b**) 90 min (**c**) 180 min (**d**) 270 min.

The cross-sectional SEM images of a hydrothermally prepared ZnO nanostructure on AZO with 40 nm Al is shown in Figure 6 with different growth times. The growth of the mentioned ZnO nanostructures occurred for 45 min, 90 min, 180 min and 270 min, respectively, as shown in Figure 6a–d. The reduction in ZnO growth size with the growth time of 270 min may suggest that 270 min was an excessive amount of time for ZnO hydrothermal synthesis in the current study. All of the samples mentioned in the figure were annealed at 500 °C.

The development of the ZnO layer was conducted over an aluminium substrate of 100 nm thickness with different growth times. In the process of hydrothermal synthesis, Al reacted with the ZnO solution to form AlO_2_^-^ and Zn^2+^. Studies indicated that the growth rate of the ZnO nanostructure depends upon the seed layer (which is aluminium); increases in the seed layer (Al) increase the growth rate of the ZnO structure [26]. From the cross-sectional images, it can be seen that the seed layer used in our study dissolved in the solution during hydrothermal synthesis (with minimal traces of Al particles present in the structure), which in turn developed the ZnO nanostructure.

**Figure 7:**
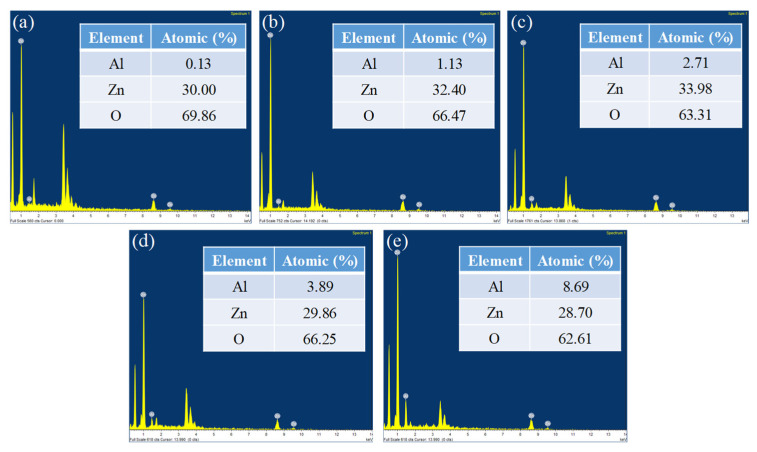
EDS spectrum analysis of (**a**) Pure AZO, (**b**) AZO with 10 nm of Al, (**c**) AZO with 20 nm of Al, (**d**) AZO with 40 nm of Al, (**e**) AZO with 80 nm of Al.

The Energy Dispersive Spectroscopy (EDS) analysis of different ZnO nanostructures with different AZO and Al compositions is shown in Figure 7. The EDS spectrum analysis in Figure 7 provides the composition of the elements of all the ZnO nanostructures in terms of their atomic weight percentages. Figure 7a shows a sample of ZnO nanostructure developed on pure AZO and the composition was recorded to be 30% Zn, 69.86% oxygen and 0.13% Al. As the samples were coated with Al of different thicknesses, the composition of Al varied in Figure 7b–e. It was found that 10 nm of Al with AZO had a 1.13% Al atomic weight percentage, 20 nm of Al with AZO had a 2.71% Al atomic weight percentage, 40 nm of Al with AZO had a 3.89% Al atomic weight percentage and 80 nm of Al with AZO had a 8.69% Al atomic weight percentage.

From the data, it is clear that Zn and O were the key components and none of the impurities were found within the traceable limits of the EDS spectrum. High peaks of Zn and O confirmed that the produced nanoparticle was in its highest purified form [27]. As the AZO surface was coated with aluminium, the oxygen levels that resulted from the EDS spectrum should be interpreted carefully as the aluminium thickness level may have obscured the oxygen content present in AZO.

**Figure 8:**
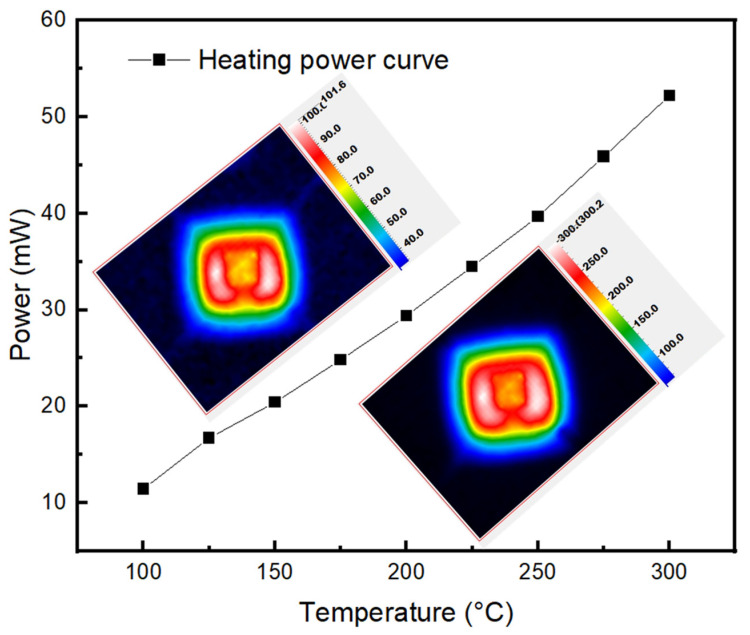
Power vs. Temperature graph of the MEMS microheater.

Power vs. Temperature graphs of the MEMS microheater were obtained under different temperatures and the data are shown in the Figure 8. The power supplied to the MEMS device was calculated by the Voltage and Ampere readings. At 11.43 mW (1.27 V and 9 mA) power, the temperature was recorded to be 100 °C. As the power increased, the temperature increased linearly with respect to power. At 52.2 mW (2.90 V and 18 mA) power, the temperature was measured to be 300 °C. The inset Figure shows the thermal images of MEMS microheater at 100 °C and 300 °C.

**Figure 9:**
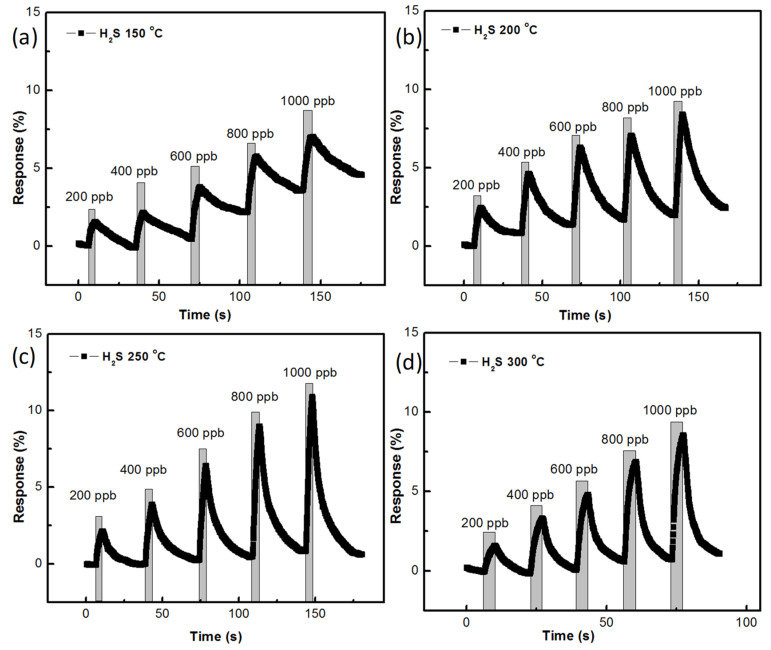
H_2_S gas-sensing response of ZnO nanostructure prepared with 45 min growth time: (**a**) at 150 °C, (**b**) 200 °C, (**c**) 250 °C, (**d**) 300 °C.

Gas-sensing responses of the ZnO sensor, prepared with AZO and 40 nm Al thickness, at different temperatures are shown in Figure 9a–d. The growth time of 45 min was used with the mentioned samples of ZnO, as can be seen in Figure 9. H_2_S gas sensing was measured at concentrations from 200 ppb to 1000 ppb with differences of 200 ppb. At 150 °C, the gas-sensing responses were recorded to be 1.57% for 200 ppb of H_2_S gas and 7.02% for 1000 ppb of H_2_S gas. It was noted that the recovery of H_2_S was expected to be greatly improved at 150 °C. At a temperature of 200 °C, a 2.47% sensing response was recorded for 200 ppb of H_2_S gas and an 8.40% sensing response was recorded for 1000 ppb of H_2_S gas. The recovery of H_2_S at 200 °C was also noted to be unstable. At 250 °C, a 2.13% H_2_S gas-sensing response for 200 ppb and a 10.92% H_2_S gas-sensing response for 1000 ppb were recorded. The recovery of H_2_S gas was noted to be stable at 250 °C. At 300 °C, a 1.58% H_2_S gas-sensing response for 200 ppb and an 8.55% H_2_S gas-sensing response for 1000 ppb were recorded and the recovery of H_2_S was also stable.

**Figure 10:**
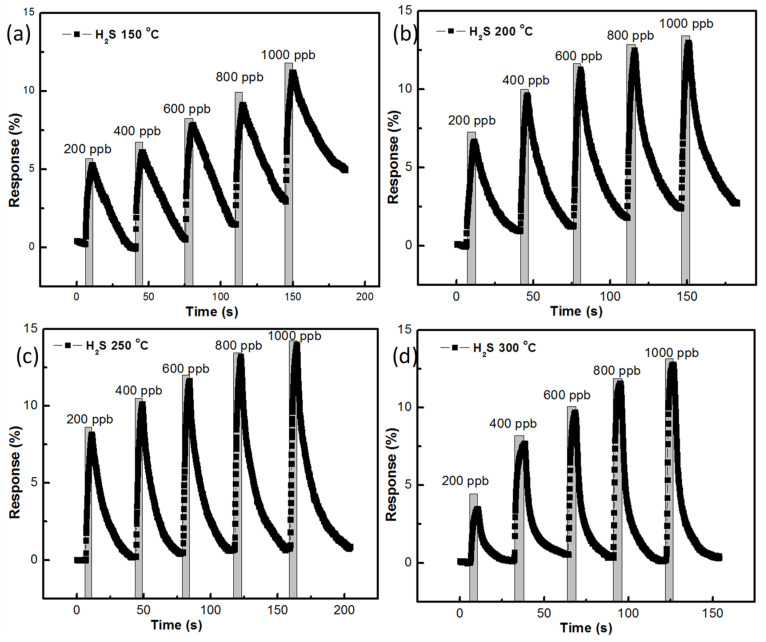
H_2_S gas-sensing response of ZnO nanostructure prepared with 90 min growth time: (**a**) at 150 °C, (**b**) 200 °C, (**c**) 250 °C, (**d**) 300 °C.

Gas-sensing responses of the ZnO sensor, prepared with AZO and Al of 40 nm thickness at different temperatures, are shown in Figure 10a–d. The growth time of the ZnO nanostructure was 90 min. At 150 °C, the sensing responses of H_2_S were recorded and are shown in Figure 10a, where it can be seen that the recovery of the gas did not reach the base point. A quantity of 200 ppb of H_2_S had a 5.287% sensing response while 1000 ppb of H_2_S gas showed a 11.203% sensing response. At 200 °C, 200 ppb of H_2_S had a 6.664% sensing response while 1000 ppb of H_2_S gas had a 13.005% sensing response. At 250 °C, 200 ppb of H_2_S gas had a 8.143% sensing response while 1000 ppb of H_2_S gas had a 13.991% sensing response with a good recovery curve. At 300 °C, the sensing responses dropped from that of 250 °C, as a 3.485% sensing response was recorded for 200 ppb of H_2_S and a 12.801% sensing response was recorded for 1000 ppb of H_2_S.

**Figure 11:**
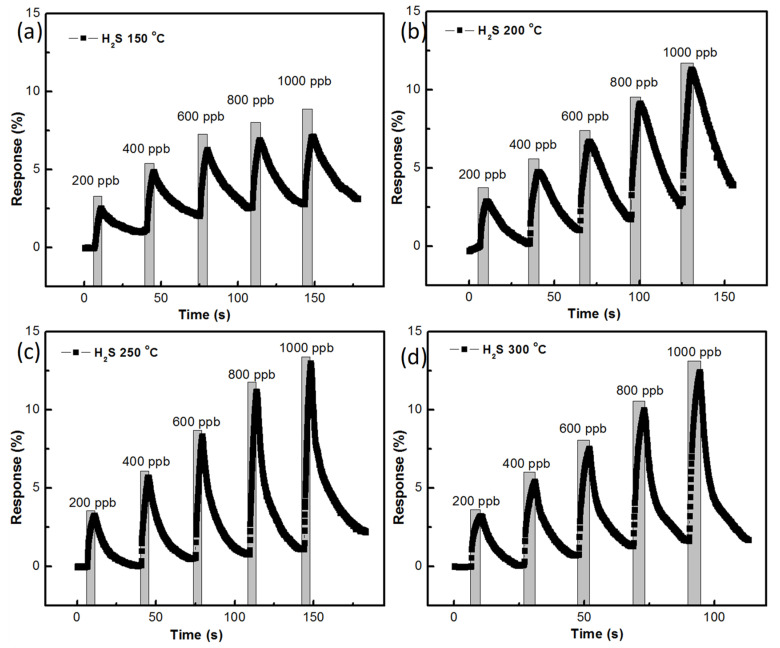
H_2_S gas-sensing response of the ZnO nanostructure prepared with 180 min growth time: (**a**) at 150 °C, (**b**) 200 °C, (**c**) 250 °C, (**d**) 300 °C.

The gas-sensing responses of the ZnO sensor, prepared with AZO and Al of 40 nm thickness at different temperatures, are shown in Figure 11a–d. The growth time of the ZnO nanostructure was 180 min. At 150 °C, 200 ppb of H_2_S gas had a 2.533% sensing response and 1000 ppb of H_2_S gas had a 7.14% sensing response. At 200 °C, 200 ppb of H_2_S gas had a 2.89% sensing response while 1000 ppb of H_2_S gas had a 11.288% sensing response. At 250 °C, for 200 ppb of H_2_S gas, the sensing response was recorded to be 3.247%. and for 1000 ppb of H_2_S gas, the sensing response was recorded to be 12.988%. At 300 °C, the sensing response of H_2_S gas at 200 ppb was 3.196%, and was 12.41% at 1000 ppb.

Temperature has a prominent role in terms of the results of gas sensing with semiconductor metal oxides. The optimal temperature of the sensor depends upon the sensing material, the dopant and the method of synthesis [28]. In the current study, different temperature gas-sensing tests were conducted with different growth times to identify the optimal sensing temperature of the AZO-doped ZnO sensor for H_2_S gas sensing. When the H_2_S (reducing) gas came into contact with the ZnO (n-type) sensor, the resistance decreased and the value of the resistance changed depending on the temperature. In gas sensing, different temperatures induce different levels of conductivity, which in turn shows different resistance values for the same gas concentration [29]. In Figure 9, Figure 10 and Figure 11, the results obtained from measuring the change in the resistance at different temperatures are shown. All of the sensing results showed a good amount of change in resistance when the H_2_S gas entered the chamber. However, to pinpoint the optimal temperature, two parameters were considered in this study. One involved identifying the highest sensing response offered by the sensor at a particular temperature. The second parameter involved checking that the conductivity or resistance curve return to the original value. From the data acquired, at 150 °C and 200 °C, the change in resistance and conductivity was not complete, i.e., the conductivity and resistance curves did not return to their original positions. In addition, the sensing responses were very low compared to those obtained at 250 °C and 300 °C. For the 250 °C and 300 °C temperature parameters, the sensing response was high and the conductivity and resistance curves showed great recovery responses. However, the 250 °C temperature variant showed a better response than the 300 °C temperature variant.

**Figure 12:**
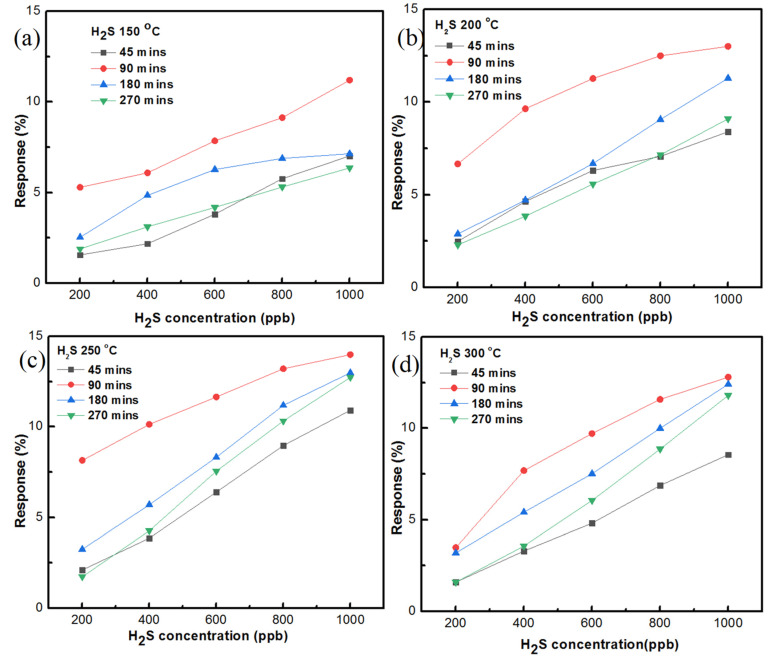
Summary of the H_2_S gas-sensing responses of ZnO nanostructure prepared on AZO and 40 nm Al at different temperatures with growth times of (**a**) 45 min, (**b**) 90 min, (**c**) 180 min, (**d**) 270 min.

The gas-sensing responses of the ZnO sensor, prepared with AZO and Al of 40 nm thickness at different temperatures, are shown in Figure 12a–d. The growth time of the ZnO nanostructure was 270 min. At 150 °C, the gas sensing of H_2_S gas at 200 ppb had a 1.887% response and 1000 ppb of H_2_S gas had a 6.358% sensing response. At 200 °C, 200 ppb of H_2_S gas had a 2.295% sensing response and 1000 ppb of H_2_S gas had a 9.095% sensing response. At 250 °C, a 1.734% sensing response was recorded for 200 ppb of H_2_S while a 12.733% sensing response was recorded for 1000 ppb of H_2_S gas. At 300 °C, the sensing response reached 1.598% for 200 ppb of H_2_S gas while the sensing response reached 11.798% for 1000 ppb of H_2_S gas.

The summary of the H gas-sensing responses of the ZnO sensor prepared on AZO and 40 nm Al is shown in Figure 12 with different temperatures and different growth times. Of all the growth times, the 90 min growth time reached the highest sensing response at 250 °C, which was 13.991% with 1000 ppb of H_2_S gas. In addition, taking into account all of the Figures, it can be seen that 90 min growth time dominated every other condition to be the ideal growth time condition. The second-best sensing responses were recorded at 180 min growth time and the sensing response reached to 12.998% at 250 °C. For the ideal temperature, at 250 °C the sensing responses were recorded as being high in every growth time condition. Thus, based on the available data, it is considered that 250 °C is the ideal temperature condition for the current study. Furthermore, the 150 °C temperature sensing responses were found to be lower than other temperature variants. In terms of growth time, a low H_2_S gas-sensing response was recorded for the 45 min growth condition with the ZnO sensor. From the summary of the sensing responses, it is clear that the ZnO sensor prepared with 90 min growth time and at 250 °C had superior H_2_S gas sensing, among other conditions.

The response and recovery of the gas sensing depends on the adsorption and desorption of gas molecules at a temperature variant. The diffusion of gas molecules is directly proportional to temperature until the optimal temperature is reached, which can accelerate the diffusivity of the sensing gas molecules on the metal oxide surface, and this can lead to a good response function. When there was an increase in temperature beyond the optimal point, the response started to decrease due to desorption process, which decreased the amount of H_2_S gas molecules on the sensor surface [30]. Thus, it is important to find out the optimal temperature of the respective sensor. From the summary Figures shown in Figure 12, it can be seen that the best response and recovery occurred at 250 °C, which was the optimal temperature for the AZO-doped ZnO nanostructure in the current study. The gas-sensing response was calculated using the formula S% = (Ra − Rg/Rg) × 100. In this formula, Ra is the resistance of the sensor in the presence of air, and Rg is the resistance of the sensor in the presence of gas.

**Figure 13:**
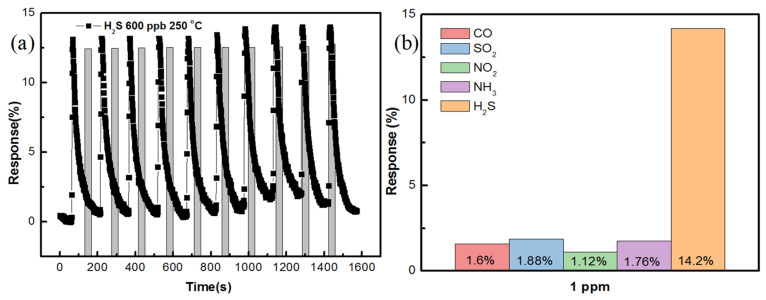
(**a**) Repeatability gas-sensing test; (**b**) selectivity of ZnO sensor for different target gases.

A random repeatability test of the ZnO sensor with AZO and 40 nm Al was conducted with H_2_S gas at 600 ppb concentration. The results of the repeatability test are shown in Figure 13a. The growth time of the sensor was 90 min and the temperature was 250 °C. A quantity of 600 ppb of H_2_S gas was tested multiple times and the sensing response remained constant during each test. The selectivity of the sensor was also tested with five different gases such as CO, SO_2_, NO_2_, NH_3_ and H_2_S at the same gas concentrations. H_2_S gas showed tremendous selectivity over other gases and the results are shown in Figure 13b. As mentioned, the operating temperature was very important in terms of the gas sensing and selectivity of a particular gas. Every gas had its own optimal temperature for a particular semiconductor metal oxide sensor. The same phenomenon was tested by Bruno et al. [31]; in their study, two reductive gases, such as CO and NO_2_, were tested at the same temperature with the same sensor, and one gas showed high selectivity than the other. Furthermore, when the temperature was reduced, the second gas showed higher selectivity than the first gas. In our study, H_2_S gas showed good sensing responses at 250 °C and even though the sensor exhibited responses to other gases, H_2_S gas had better selectivity as compared to the other gases recorded in the results.

**Figure 14:**
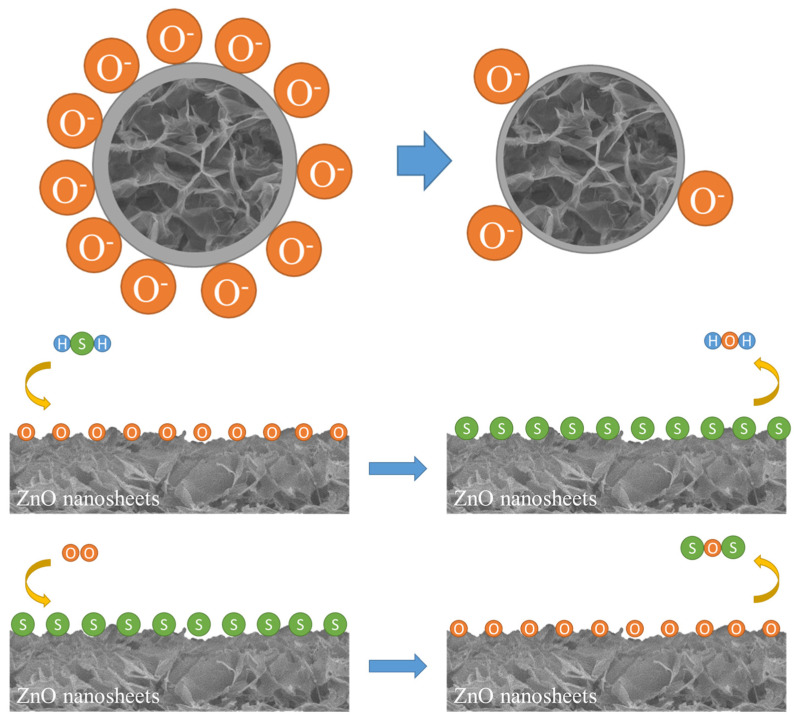
Gas-sensing mechanism of ZnO sensor with H_2_S gas.

The gas-sensing mechanism of the ZnO sensor with H_2_S gas is shown in Figure 14. Since ZnO is an n-type semiconductor, when exposed to air, the oxygen molecules were adsorbed on the ZnO surface and formed oxygen ions (O^−^), as outlined in Equation (1). This process is called chemical adsorption, and it resulted in the formation of a carrier depletion layer, resulting in a decrease in the conductivity and a simultaneous increase in the resistance of the semiconductor.

(1) $$O_{2}+2e^{-}\to 2O_{2}^{-}$$

(2) $$2H_{2}S+3O_{2}^{-}\to 2H_{2}O+{\mathrm{SO}}_{2}+3e^{-}$$

(3) $$\mathrm{ZnO}+H_{2}S\to \mathrm{ZnS}+2H_{2}O$$

(4) $$2\mathrm{ZnS}+3O_{2}\to 2\mathrm{ZnO}+2{\mathrm{SO}}_{2}$$

In the presence of H_2_S gas, oxygen ions (O_2_^−^) reacted with H_2_S gas to form H_2_O and release electrons (e^−^), as shown in Equation (2), which returned to the ZnO semiconductor. This process is called desulphurization. Then, the sulphur molecules were absorbed by the ZnO surface and formed ZnS, as shown in Equation (3). At this point, the resistance of the semiconductor dropped.

When the semiconductor was brought back to the presence of air, the sulphur molecules were desorbed and ZnS was converted back to ZnO, as shown in Equation (4). When this phenomenon occurred, the resistance returned to the original point.

The gas-sensing responses of different research works are compared in Table 1, as shown below. Different combinations of the ZnO nanostructure were observed in the literature, and each nanostructure was different and unique. It was observed that each structure had different optimal temperatures for gas sensing due to the variation in the synthesis process and parameters. The current study found the optimal temperature to be 250 °C with the highest response of 14% recorded for 1 ppm H_2_S gas.

## 4. Conclusions

By means of hydrothermal synthesis, ZnO nanostructures were successfully developed on AZO with Al of different thicknesses. The structural properties of ZnO were determined by XRD, SEM and EDS analysis. The thermal properties of the MEMS microheater were calculated using thermal images. The sensing properties of the ZnO sensor prepared on AZO with 40 nm Al were determined with different growth times and different temperatures. From the results, it is understood that the conditions of 90 min growth time and 250 °C temperature led to the best performance in terms of sensing responses with H_2_S gas. The sensing response reached 14% with 1 ppm (1000 ppb) of H_2_S gas. The selectivity of ZnO was assessed with five different gases, namely CO, SO_2_, NO_2_, NH_3_ and H_2_S, and the sensor showed better selectivity for H_2_S gas than other gases.

## Figures and Tables

**Table 1:** Comparison of current research with previous works.

Material and Nanostructure	H_2_S Gas Concentration	Operating Temperature	Gas-Sensing Response	References
CuO-ZnO hollow spheres	5 ppm	336 °C	13.3%	[32]
CuO-ZnO nanoparticle	2 ppm	225 °C	10%	[33]
NiO-ZnO nanorods	20 ppm	160 °C	21.3%	[34]
ZnTe-ZnO nanorods	100 ppm	200 °C	70%	[35]
AZO-/Al-based ZnO nanosheets	1 ppm	250 °C	14%	This research

## Data Availability

The data used to support the findings of this research are available upon request.
